# A novel method to retrieve alerts from a homegrown Computerized Physician Order Entry (CPOE) system of an academic medical center: Comprehensive alert characteristic analysis

**DOI:** 10.1371/journal.pone.0246597

**Published:** 2021-02-09

**Authors:** Shuo-Chen Chien, Yen-Po (Harvey) Chin, Chang Ho Yoon, Md. Mohaimenul Islam, Wen-Shan Jian, Chun-Kung Hsu, Chun-You Chen, Po-Han Chien, Yu-Chuan (Jack) Li

**Affiliations:** 1 Graduate Institute of Biomedical Informatics, College of Medical Science and Technology, Taipei Medical University, Taipei, Taiwan; 2 International Center for Health Information and Technology, College of Medical science and Technology, Taipei Medical University, Taipei, Taiwan; 3 Department of Biomedical Informatics, Harvard Medical School, Boston, Massachusetts, United States of America; 4 Department of Medicine, Brigham and Women’s Hospital and Harvard Medical School, Boston, Massachusetts, United States of America; 5 School of Health Care Administration, Taipei Medical University, Taipei, Taiwan; 6 Information Technology Office, Wan Fang Hospital, Taipei Medical University, Taipei, Taiwan; 7 Department of Radiation Oncology, Wan Fang Hospital, Taipei Medical University, Taipei, Taiwan; 8 Department of Business Administration, National Taiwan University, Taipei, Taiwan; 9 Department of Dermatology, Wan Fang Hospital, Taipei Medical University, Taipei, Taiwan; University of Sydney, AUSTRALIA

## Abstract

**Background:**

The collection and analysis of alert logs are necessary for hospital administrators to understand the types and distribution of alert categories within the organization and reduce alert fatigue. However, this is not readily available in most homegrown Computerized Physician Order Entry (CPOE) systems.

**Objective:**

To present a novel method that can collect alert information from a homegrown CPOE system (at an academic medical center in Taiwan) and conduct a comprehensive analysis of the number of alerts triggered and alert characteristics.

**Methods:**

An alert log collector was developed using the Golang programming language and was implemented to collect all triggered interruptive alerts from a homegrown CPOE system of a 726-bed academic medical center from November 2017 to June 2018. Two physicians categorized the alerts from the log collector as either clinical or non-clinical (administrative).

**Results:**

Overall, 1,625,341 interruptive alerts were collected and classified into 1,474 different categories based on message content. The sum of the top 20, 50, and 100 categories of most frequently triggered alerts accounted for approximately 80, 90 and 97 percent of the total triggered alerts, respectively. Among alerts from the 100 most frequently triggered categories, 1,266,818 (80.2%) were administrative and 312,593 (19.8%) were clinical alerts.

**Conclusion:**

We have successfully developed an alert log collector that can serve as an extended function to retrieve alerts from a homegrown CPOE system. The insight generated from the present study could also potentially bring value to hospital system designers and hospital administrators when redesigning their CPOE system.

## Introduction

In the United States, medication errors kill 7,000 to 9,000 people every year, and the total cost per year of looking after patients with medication-associated errors exceeds $40 billion [1]. Several studies have verified that the implementation of alerts in Computerized Physician Order Entry (CPOE) systems can effectively prevent medication errors, such as prescriptions for the wrong patient and intrinsic adverse drug events, resulting in improved prescriber behavior and/or patient outcomes [2–4].

Interruptive alerts, in the form of suspended pop-up windows, have emerged as an effective way to prevent dangerous overrides when compared to soft-stop and passive alerts. In contrast to interruptive alerts, soft-stop and passive alerts can be ignored by the physician. Although interruptive alerts from the CPOE system may ensure that physicians see them, an excessive number of alerts that add little value to the clinical decision-making process could lead to unintended consequences, such as additional cognitive burden, and delaying the delivery of appropriate therapy [5–7].

Alert fatigue, defined as fatigue resulting from a high frequency of nonactionable and false alarms, may potentially cause physicians to ignore important clinical alerts and lead to medical error [8, 9]. Several observational and interventional studies have reported that approximately 49%-96% of alerts were overridden [8]. The reasons that cause alert fatigue have been proposed by different researchers, and include 1) irrelevant alerts; 2) a failure to communicate the actual meaning of the alert; and 3) a large number of alerts [10–12]. In addition to clinical alerts, administrative (non-clinical) alerts also comprise an important part of alerts on the CPOE system.

The most direct way to reduce alert fatigue is to remove alerts of low-clinical value in the CPOE system. In order to achieve this goal, understanding the types and distribution of alert categories within a hospital are necessary, and can be achieved using an alert log collector. While an alert log collector might be part of the commercially-available CPOE system, it may be missing in some homegrown CPOE systems. Therefore, the first objective of this study was to develop an alert log collector that was compatible with our homegrown CPOE system. Moreover, previous studies have only focused on clinical alerts [3, 13, 14]; non-clinical alerts have rarely been described. To the best of our knowledge, there is currently no study assessing the distribution of clinical and non-clinical types of alert. We hypothesized that most of the interruptive alerts would be clinically related, as this would be a more logical design choice for any clinical alert system. After the alert log collector was established, we aimed to examine the distribution of different alert types at an academic medical center in Taiwan.

## Methods

### Hospital characteristics

Taipei Municipal Wan Fang Hospital (WFH), an academic medical center managed by Taipei Medical University, was established on February 15, 1997. This hospital has 726 beds and has been using a homegrown CPOE system since 1997. WFH first obtained Joint Commission International Accreditation (JCIA) on July 21, 2006, and became the first academic medical center in Taiwan to receive it. To date, WFH has passed JCI accreditation three times, demonstrating its ability to provide patient care while satisfying internationally-recognized standards. This is a retrospective study, and it meets the IRB review exemption criteria of our organization because the data cannot be linked to any human subjects. Data are available from the Wan Fang Hospital Institutional Data Access for researchers by application. If readers are interested in the original dataset, please contact yianyulin@w.tmu.edu.tw (Miss Yian-Yu Lin).

### The implementation of an alert log collector function for the CPOE system

**Fig 1:**
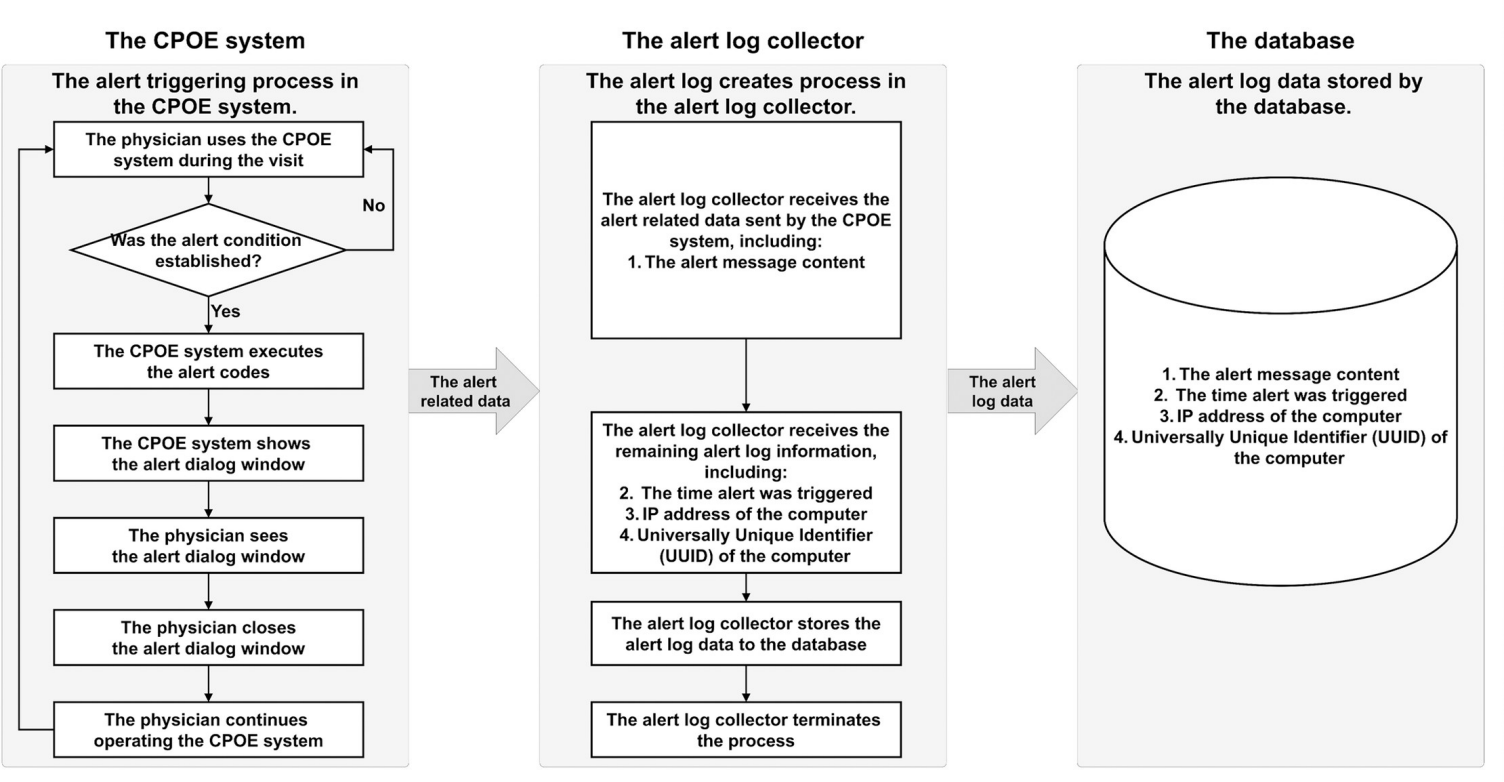
Framework of alert log collector. (CPOE = Computerized Physician Order Entry).

The Go (Golang) programming language (Version 1.9) was used to build the alert log collector function, which operates independently from the hospital’s CPOE system. The database was maintained with MySQL. The original source code can be found on GitHub using the following link: https://github.com/alanjian/AlertLogCollector. The alert log collector was implemented in the CPOE at WFH from 11 November, 2017, to 22 June, 2018. A total of 1,625,341 alerts were collected over 224 days, of which 27 days were missing due to internet disconnection or server downtime upgrades. The operating framework of the alert log collector in the CPOE system is shown in Fig 1.

When an alert was triggered, the CPOE system popped up an alert window containing the message on the computer screen. All of the alerts from our CPOE system were collected by the alert log collector running in the background of the physician’s computer, and the alert information was transmitted to our database for analysis.

### Alert characterization and expert review

**Fig 2:**
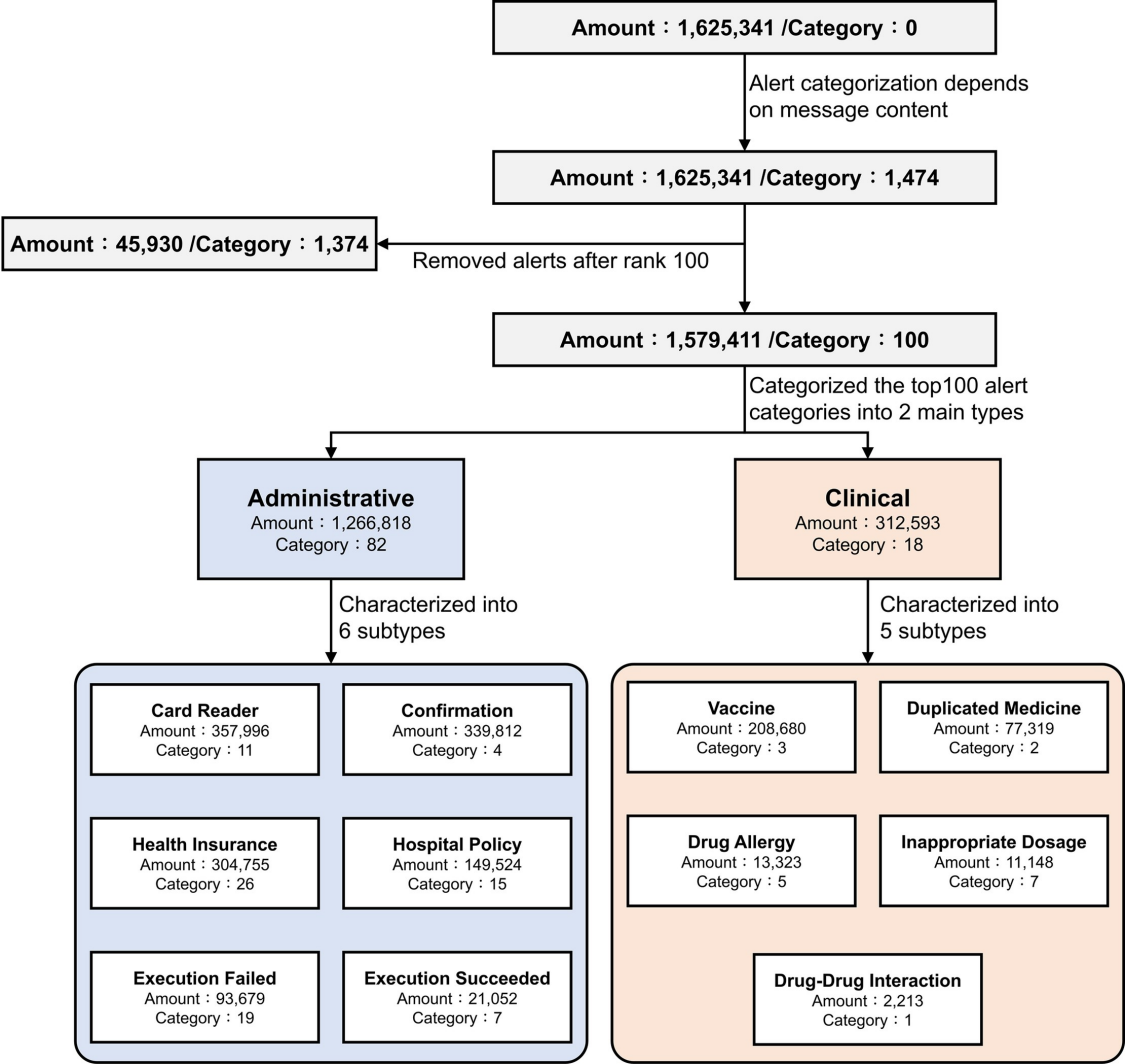
Alert characterization framework.

The alert characterization framework is as shown in Fig 2. Of the 1.6 million alerts we originally collected, some of the messages related to the same category of alert but were expressed in more messages than required: for example, "[Drug A] has been suspended. Do you want to use [Drug B] as an alternative option?" The different drug names "[Drug A]" and "[Drug B]" appeared in separate alert messages i.e. two independent alerts. Thus, based on alert message content, we characterized 1,625,341 alerts into 1,474 different categories. Both alert types—clinical and non-clinical—appeared on the physician’s screen. The top 100 most frequently triggered alert categories (1,579,411 alerts) were identified and further classified as clinical or non-clinical (administrative) by two physicians.

We identified six different subtypes of non-clinical (administrative) alerts: "Card Reader", "Confirmation", "Health Insurance", "Hospital Policy", "Execution Failure" and "Execution Succeeded". We identified five subtypes of clinical alerts: “Vaccine”, “Duplicate Medicine”, “Drug Allergy”, “Inappropriate Dosage” and “Drug-Drug Interaction”. The detailed definitions of each alert subtype are listed below:

1. Administrative:
  1.1 Card Reader: This type of alert is related to the health insurance card reader, which includes “card not inserted”, “card not detected”, “incorrect card”.
  1.2 Confirmation: This type of alert serves as a confirmation that the CPOE system has successfully executed the user’s order.
  1.3 Health Insurance: This type of alert is related to health insurance declarations.
  1.4 Hospital Policy: This type of alert is related to hospital policy announcements, and directives that physicians must follow.
  1.5 Execution Failed: This type of alert is generated when the CPOE system cannot complete the instructions issued by the user.
  1.6 Execution Succeed: This type of alert is generated when the CPOE system successfully completes the instructions issued by the user.
2. Clinical:
  2.1 Vaccine: This type of alert is related to vaccines and infectious disease notifications.
  2.2 Duplicated Medicine: This type of alert is generated when another drug with the same active chemical or pharmacological mechanism was prescribed on the same day or at the same time.
  2.3 Drug Allergy: This type of alert is generated if a patient develops an allergic response to the drug.
  2.4 Inappropriate Dosage: This type of alert is generated if the dosage of the drug is not within the normal range of prescribing doses.
  2.5 Drug-Drug Interaction: This type of alert is generated if at least two drugs on the patient’s medication list are known to affect one another.

Two physicians (YC Li, CY Chen), who are also experts in the field of computer science, were invited to review and categorize alerts as clinical or non-clinical using the different subtypes mentioned above.

### Data analysis

MySQL Relational Database Management System (RDBMS) was used to store and manage all data. The frequency of alerts triggered each day was stored. Comparisons among different types of alert were presented as counts with percentages. Cohen’s kappa coefficient (K) statistic was applied to measure the inter-rater agreement of physicians in terms of alert classification. Microsoft Excel 2016 v14.5.5 (Redmond, WA, USA) was used to compute counts, percentages, and other descriptive statistics.

## Results

### Alert distribution

We successfully developed an alert log collector using the Golang programming language, compatible with the homegrown CPOE system of an academic medical center in Taiwan. With this alert log collector, a total of 1,625,341 triggered alerts were collected in the 8-month period (from November 11, 2017, to June 22, 2018). Table 1 shows the distribution of alerts among different weekdays. Approximately 10,000 alerts were generated per day at WFH. The maximum and minimum numbers of alerts triggered per day from Monday to Friday were around 14,000 and 8,200, respectively. Thursday was the least represented day because the routine maintenance of servers mostly occurred on that day. Since the outpatient clinic did not operate on Sundays, we did not include Sunday for analysis.

**Table 1:** Distribution of alerts on different days of the week.

Week	Days	Mean	SD.(σ)	MAX.	MIN.
**Monday**	29	11,861.3	2,251.9	15,399	6,781
**Tuesday**	29	10,742.0	1,590.1	14,086	7,959
**Wednesday**	28	11,240.0	1,581.3	14,468	8,966
**Thursday**	25	10,778.4	1,571.1	13,336	8,398
**Friday**	26	10,702.6	1,306.5	13,013	8,899
**Saturday**	26	4,606.0	731.7	6,121	3,505

Abbreviations: SD. = Standard deviation, MAX. = Maximum, MIN. = Minimum

The homogeneity test (Levene test) showed a statistically significant difference between the amount of alerts on different days of the week. Thus, the post-hoc Dunnett T3 test was conducted to compare the number of alerts on different days of the week (Table 2). There was no statistical significance between the days from Monday to Friday. However, there was a significant difference (P<0.05) between Saturday and every other day. The reason was that the outpatient clinics only operated for half the day on Saturdays. This also indicated that the number of alerts was potentially related to the amount of outpatient visits.

**Table 2:** Dunnett T3 test between the amount of triggered alerts and different days of the week.

(I) Week	(J) Week	MD(I-J)	SE	P	95% Confidence Interval	
					Lower Bound	Upper Bound
Monday	Tuesday	1119.241	511.918	0.379	-449.11	2687.59
	Wednesday	621.24	513.987	0.973	-953.54	2196.02
	Thursday	1082.876	523.073	0.463	-520.23	2685.98
	Friday	1158.699	490.437	0.273	-350.73	2668.13
	Saturday	7255.314*	442.116	0	5872.11	8638.51
Tuesday	Wednesday	-498.001	420.113	0.978	-1780.22	784.21
	Thursday	-36.366	431.181	1	-1356.6	1283.87
	Friday	39.458	390.949	1	-1155.94	1234.86
	Saturday	6136.073*	328.296	0	5118.78	7153.37
Wednesday	Thursday	461.636	433.635	0.991	-866.83	1790.11
	Friday	537.459	393.654	0.932	-667.53	1742.45
	Saturday	6634.074*	331.513	0	5604.39	7663.76
Thursday	Friday	75.823	405.445	1	-1170.75	1322.4
	Saturday	6172.438*	345.432	0	5090.02	7254.86
Friday	Saturday	6096.615*	293.679	0	5185.28	7007.95

Note

* = P ≤ 0.05

### Ranking of top 100 alerts

**Fig 3:**
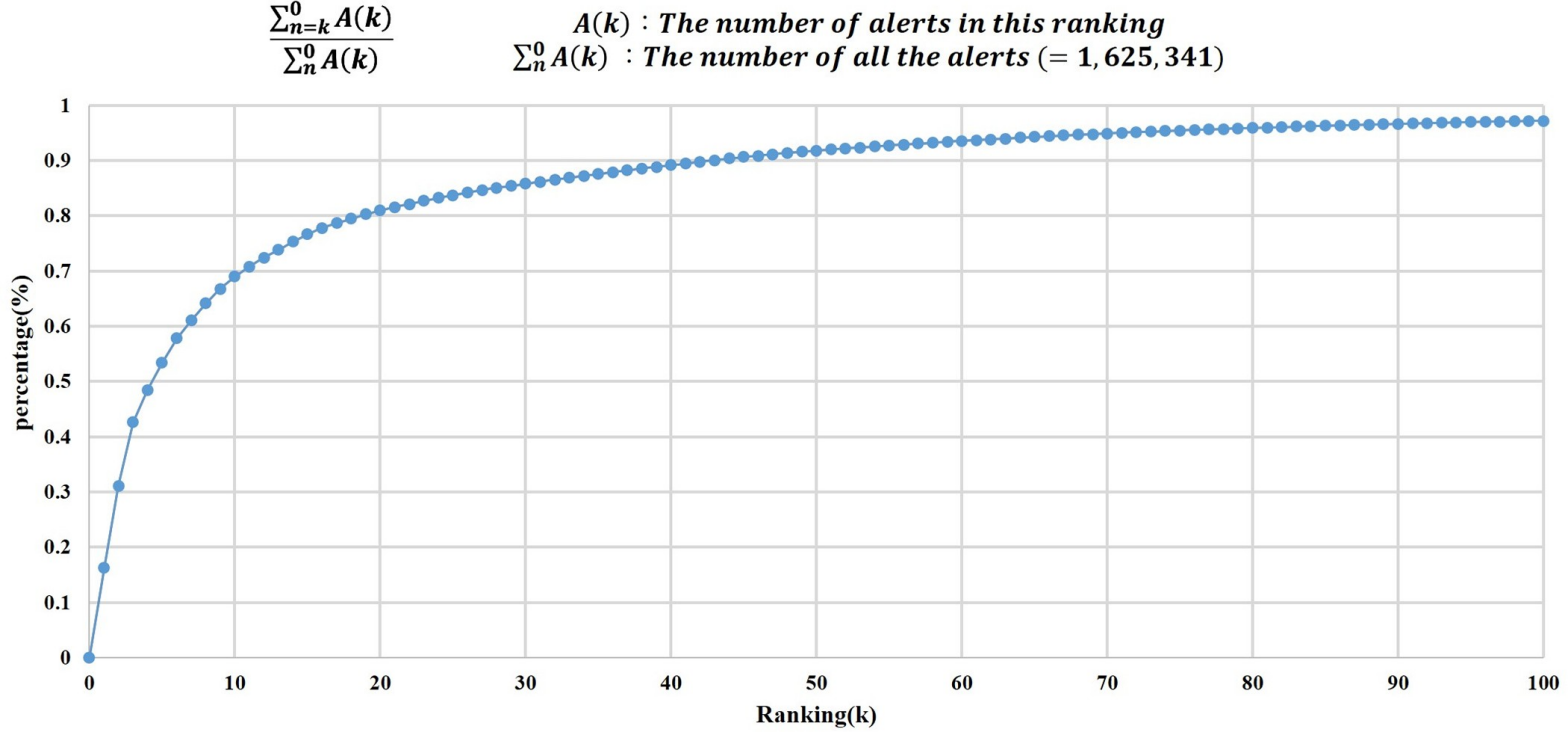
Cumulative distribution of top 100 alerts.

There were 1,474 different types of alerts based on their message content. Notably, alerts relating to the top 100 categories accounted for 97% (1,579,411/ 1,625,341 = 0.971) of the total alerts generated within the CPOE system during the study period (Fig 3). Meanwhile, the top 10, 20, and 50 categories of alerts covered 70, 80, and 90 percent of the entire alerts generated in the CPOE system, respectively. The top 20 categories of message content are displayed in Table 3.

**Table 3:** Content of the top 20 alerts triggered within the CPOE system.

#	Types		Categories (Message content of the alert)	Amount
	Main	Sub		
**1**	A.	Secondary Confirmation	Are you sure you want to cancel? It won’t save while the health insurance card was removed.	263,838
**2**	A.	Card Reader	Failed to open the online medication record, please check the card reader!	241,531
**3**	C.	Vaccine	The patient is qualified for a free flu shot covered by the NHI!	186,813
**4**	C.	Health Insurance	This is a primary healthcare diagnosis!	94,598
**5**	C.	Hospital Policy	[Drug A] has been suspended. Do you want to use [Drug B] as an alternative option?	81,155
**6**	C.	Duplicate Medicine	[Drug A] was prescribed by another physician on [YYYYMMDD] and still has [N] days of medication remaining. Do you want to continue prescribing?	71,902
**7**	A.	Card Reader	The data has been saved to the health insurance card, now you may take it out.	51,994
**8**	A.	Secondary Confirmation	Do you want to reprint the invoice?	50,639
**9**	A.	Health Insurance	Do you want to prescribe the drug at the patient’s own expense?	42,525
**10**	A.	Health Insurance	Preserve this critical illness in the problem list, automatically include this critical illness next time when entering orders!	36,204
**11**	A.	Execution Failed	An incorrect password was entered for [N] times!	29,588
**12**	A.	Health Insurance	In order to ensure the quality of medical care and reduce the OPD deducted rate of NHI, please complete the inspection chart carefully.	25,605
**13**	A.	Secondary Confirmation	Are you sure to delete all? ([Null / Chemotherapy drugs or self-check-in have been excluded])	23,537
**14**	A.	Health Insurance	The patient is qualified for child Medicaid. The validity period of the child Medicaid certificate is from [YYYMMDD] to [YYYMMDD].	23,533
**15**	A.	Card Reader	The information of Patient and IC card didn’t match. Please confirm whether the IC card is his/her card?	21,825
**16**	A.	Card Reader	The card reader is occupied, please try to finish the observation later.	18,385
**17**	A.	Vaccine	The International Disease Code [ICD 9 or ICD 10] has already existed.	14,566
**18**	A.	Health Insurance	A diagnosis of chronic disease is required if the prescription is for more than 7 days.	13,393
**19**	A.	Hospital Policy	Do you want to update the patient’s information?	12,963
**20**	A.	Execution Failed	Failed to set the printer!	10,928

Note: A. = Administrative, C. = Clinical

(CPOE = Computerized Physician Order Entry)

### Alert characterization

**Fig 4:**
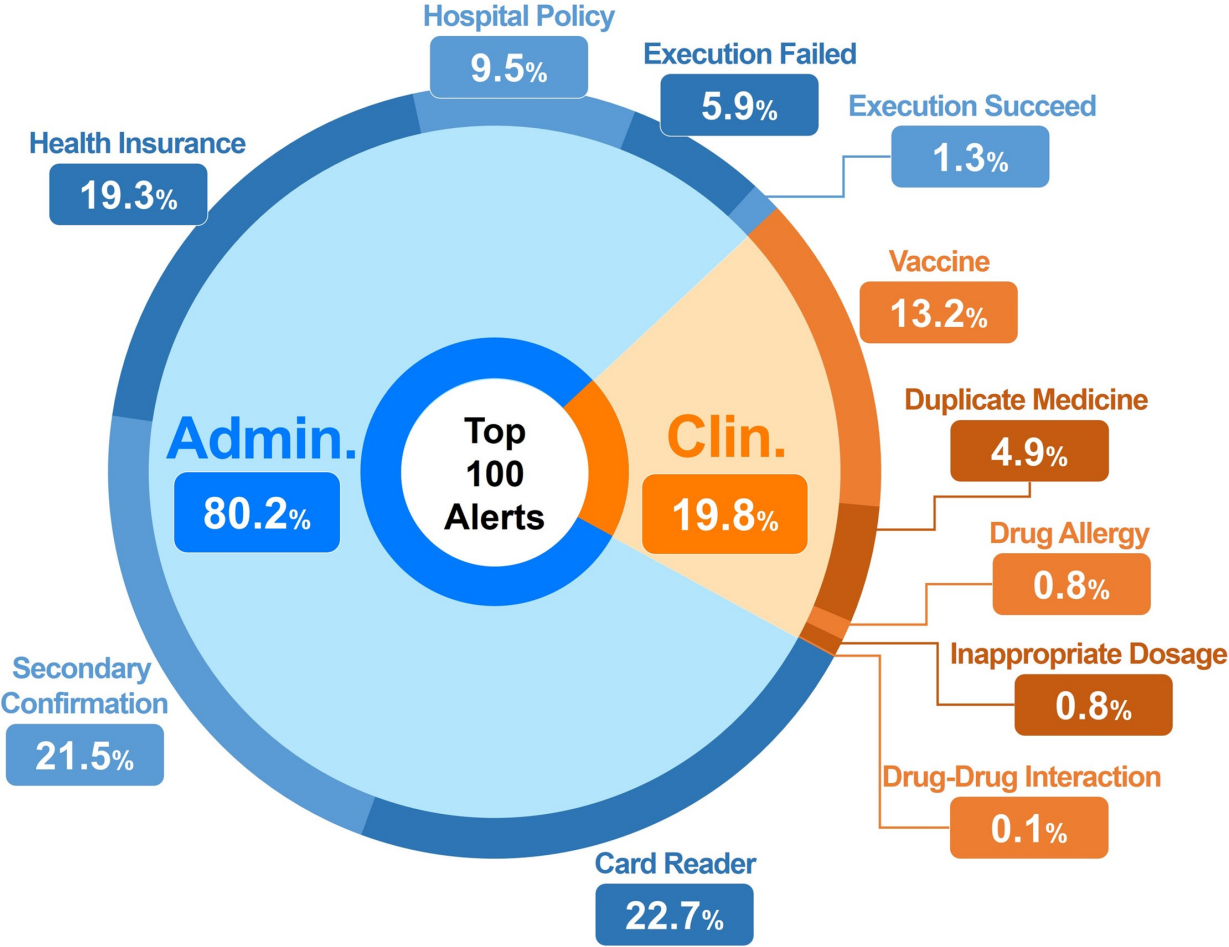
Classification of the top 100 alerts. (Admin. = Administrative, Clin. = Clinical).

The top 100 categories of message content were further categorized as non-clinical (administrative) alerts or clinical alerts. There was good inter-rater agreement for classifying alerts as clinical or non-clinical (administrative): kappa = 0.997; 95% confidence interval, 0.993 to 1.0. Non-clinical administrative alerts accounted for 80.2% of the total alerts. Of these, a total of 357,996 alerts (22.7%) were card reader-related alerts and 339,812 alerts (21.5%) were secondary confirmation alerts. The remaining 312,593 (19.8%) alerts were clinical alerts; among these alerts, 13.2% alerts were related to vaccines, 4.9% were related to duplicate medications, and 1.6% (0.8%+0.7%+0.1%) alerts were due to drug allergy (0.8%), inappropriate dosage (0.7%), and drug-drug interactions (0.1%) (Fig 4).

## Discussion

In the present study, we developed an alert log collector through the Golang programming language as an alert collection tool, which was utilized to gather and analyze the alert characteristics within the CPOE system of an academic medical center in Taiwan. According to several studies, some hospitals still utilize homegrown CPOE systems [15–17]. For such hospitals, an alert log collector may not exist. Our method can serve as a valuable tool, the source code of which is available on GitHub. Through minor modifications of CPOE source codes to incorporate the alert log collector, hospitals can add alert-collecting functions to their current homegrown CPOE system and expand their system’s ability to analyze the characteristics of their alerts. Meanwhile, this could help other hospitals understand the types and distribution of alerts generated by their CPOE system, identify potential problems, and ultimately improve the alert system.

Some studies have proved that the implementation of a CPOE system has many benefits, such as decreasing medical errors, improving physician compliance with guidelines [18], and reducing unintentional duplicate orders for laboratory tests [19]. Most CPOE systems use alerts to optimize the safety and quality of clinical decisions [20]. However, too many alerts were being generated in our CPOE system. In our study, approximately 10,000 alerts were triggered in WFH, with about 160 physicians seeing 4,800 patients per day. This means that physicians will have received two alerts on average when seeing one patient, which may have interrupted the physicians’ clinical workflow. The research done by van der Sijs H et al. in 2006, indicated that up to 96% of alerts are overridden by physicians [8]. High rates of alert recurrence and false-positive alerts [21] (irrelevant alerts) cause physicians to experience alert desensitization (alert fatigue), resulting in their ignoring important alerts that may relate to patient safety [22].

Although understanding the amount of triggered alerts in the CPOE system was important, analyzing the composition of alerts provided a different perspective to investigate the effectiveness of the alert system. Previously, a study conducted by Del et al. from the Seattle Children’s Hospital analyzed the triggered alerts within the hospital, but they only examined clinical alerts [23]. In our study, we categorized the collected alerts into clinical and administrative types in order to gain a better understanding of the distribution of triggered alerts. Our study showed that about 80 percent of the triggered alerts were administrative alerts, which are of minimal clinical value. To the best of our knowledge, this is the first study to comprehensively characterize both interruptive clinical and interruptive non-clinical alerts generated by a homegrown CPOE system within an academic medical center.

Interruptive alerts in large quantities, especially with duplicates and of low clinical value, may eventually lead to alert fatigue. One potential explanation as to why there were so many interruptive non-clinical alerts generated by the CPOE system is that the system designers may not have understood typical clinical workflows. Therefore, based on our research results, we gave the following suggestions for re-designing a CPOE alert system at WFH: first, it would be beneficial to strengthen the communication between the information technology department and clinicians. The clinical value of each alert should be re-examined and defined; alerts with similar meanings or intentions should be merged to reduce the overall number of alerts. Second, one should audit the alerts defined in the CPOE system regularly. Our research found that most of the alerts were rarely used (only 100 out of 1,479 were triggered with any frequency). A large amount of redundant programming code will make the CPOE system hard to maintain; furthermore, the alert message should be kept as short as possible while their content should be polite and easily understood by physicians [22, 24]. Third, it would be useful to remove alerts with limited clinical value. One of our main findings was that up to 80% of the alerts were clinically irrelevant. Therefore, delivering such administrative information through the same interruptive channels as clinical alerts seems suboptimal. Instead, alerts with no clinical value should be removed entirely. If they cannot be removed, soft-stop or passive-type alerts should be chosen, allowing the physician to decide whether to attend to the alert or not [22]. By following these design guidelines for the CPOE system, the medication-ordering process may be improved, decreasing alert fatigue with possible benefits to patient safety [25, 26].

### Limitations

This study has several limitations. First, although we have collected and analyzed the largest amount of alerts reported to date in a single study, our data were collected from a single academic medical center in Taiwan. This may limit the generalizability of our findings. Second, the present study uses only descriptive statistics to analyze the alert distribution; a randomized controlled trial is needed to examine the value added by the alert log collector. Lastly, the clinician acceptance rate of alerts was not recorded in this study. While we believe the insight generated from the present study can be of help when redesigning the CPOE system, a future study focusing on physicians’ behavioral changes toward the alerts might be needed to determine its true clinical value.

## Conclusion

We have successfully developed a novel method to retrieve alerts from a homegrown CPOE system. We showed that most of the alerts in the CPOE system of a single large academic medical center were administrative (non-clinical) and we provided a detailed list of the most frequently triggered alerts. Additionally, the top 100 categories of alerts by alert content accounted for 97 percent of the total number of triggered alerts. These results could potentially expand the functionality of the hospital’s homegrown system and provide valuable insights to system designers and hospital administrators when redesigning the CPOE system.
